# SynChro: A Fast and Easy Tool to Reconstruct and Visualize Synteny Blocks along Eukaryotic Chromosomes

**DOI:** 10.1371/journal.pone.0092621

**Published:** 2014-03-20

**Authors:** Guénola Drillon, Alessandra Carbone, Gilles Fischer

**Affiliations:** 1 Sorbonne Universités, UPMC Univ Paris 06, UMR 7238, Biologie Computationnelle et Quantitative, Paris, France; 2 CNRS, UMR7238, Biologie Computationnelle et Quantitative, Paris, France; 3 Institut Universitaire de France, Paris, France; Institut de Genetique et Microbiologie, France

## Abstract

Reconstructing synteny blocks is an essential step in comparative genomics studies. Different methods were already developed to answer various needs such as genome (re-)annotation, identification of duplicated regions and whole genome duplication events or estimation of rearrangement rates. We present SynChro, a tool that reconstructs synteny blocks between pairwise comparisons of multiple genomes. SynChro is based on a simple algorithm that computes Reciprocal Best-Hits (RBH) to reconstruct the backbones of the synteny blocks and then automatically completes these blocks with non-RBH syntenic homologs. This approach has two main advantages: (i) synteny block reconstruction is fast (feasible on a desk computer for large eukaryotic genomes such as human) and (ii) synteny block reconstruction is straightforward as all steps are integrated (no need to run Blast or TribeMCL prior to reconstruction) and there is only one parameter to set up, the synteny block stringency 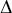. Benchmarks on three pairwise comparisons of genomes, representing three different levels of synteny conservation (Human/Mouse, Human/Zebra Finch and Human/Zebrafish) show that Synchro runs faster and performs at least as well as two other commonly used and more sophisticated tools (MCScanX and i-ADHoRe). In addition, SynChro provides the user with a rich set of graphical outputs including dotplots, chromosome paintings and detailed synteny maps to visualize synteny blocks with all homology relationships and synteny breakpoints with all included genetic features. SynChro is freely available under the BSD license at http://www.lcqb.upmc.fr/CHROnicle/SynChro.html.

## Introduction

Synteny block reconstruction consists on the identification of a series of homologous genes whose order is conserved between two (or more) genomes. Analysis of synteny conservation between different genomes allows to identify similarity patterns and differences in genome structure and content. In practice, genomes with different levels of divergence generate different types of questions and require different analysis methods and different visualization tools. For closely related genomes, synteny conservation can be performed at the DNA level, which can be useful to annotate newly sequenced genomes [1] and to identify conserved non-coding sequences [2]–[4]. For very distantly related genomes, detection of synteny conservation requires the development of statistical models or the construction of synteny profiles obtained from different genomes [5]–[7]. In this case, synteny can help to the gene annotation process based on conservation of gene clusters [6], [8] or can be used to estimate the number of whole genome duplication events [9]. For genomes sharing intermediate phylogenetic proximity, protein-coding genes may have retained enough sequence similarity and physical collinearity along chromosomes to allow synteny block reconstruction which can help infering the history of chromosomal rearrangements and the structure of ancestral genomes [10].

SynChro falls in this last category. It is designed to define conserved synteny blocks based on the relative order of protein-coding genes along chromosomes, in order to help in rearrangement and ancestral reconstruction studies. Its main properties are the followings:

1. it makes multiple pairwise comparisons and traces information shared by each pair of genomes; it is not suited to reconstruct synteny blocks shared by several genomes at a time but instead provides analysis tools to compare different sets of pairwise synteny blocks.
2. it defines syntenic homologous genes by computing protein sequence similarity (with fastp and blastp [11], [12]) and by taking into account the gene order information. It does not require to run additional tools such as blast or tribeMCL [13] prior the synteny reconstruction step (as it is the case for MCScanX [14] and i-ADHoRe [15], respectively).
3. it reconstructs synteny blocks based on syntenic homologous genes and not on DNA alignment. This enables (i) to compare both relatively close and distant genomes and (ii) in a second time, to compare the different pairwise sets of synteny blocks using genes as common denominator.
4. it allows synteny blocks to be overlapping, included in one another or duplicated, in order to (i) support comparison involving genomes having undergone a whole genome duplication event and (ii) keep the trace of small rearrangements that may be responsible for small overlaps or inclusions between synteny blocks.

SynChro is a simple algorithm that is not meant to bring new theoretical advances over existing and more sophisticated tools in the field of synteny block identification. The interests of SynChro lie in the *all in one* package with few parameters, rapid execution time and several useful visualization tools that are more flexible than that of other existing methods.

## Results and Discussion

### SynChro Algorithm

**Figure 1:**
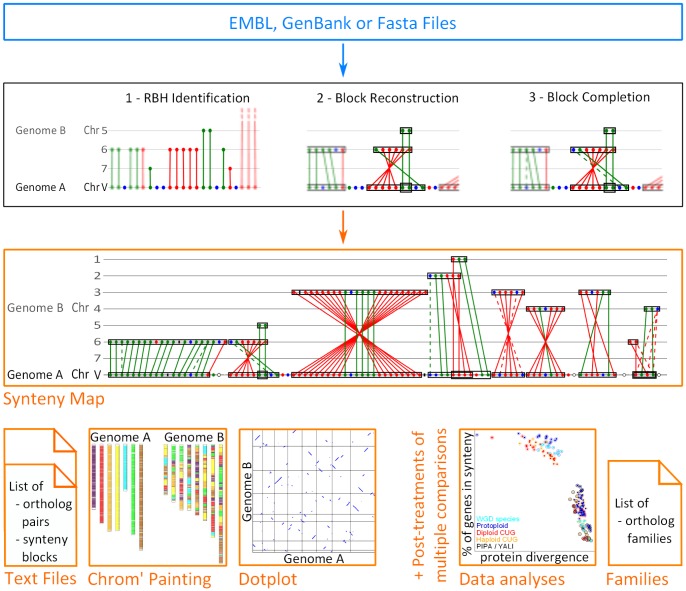
SynChro algorithm, inputs and outputs. The format of input files are indicated in the blue frame. The different steps of the algorithm are illustrated in the black frame (colored dots symbolize genes, green and red plain lines highlight RBH relationships and dotted lines represent non-RBH homologous relationships). In step 1, all RBH gene-pairs are mapped regardless of their chromosomal positions, in step 2 only the syntenic RBH-pairs are mapped and in step 3 the non-RBH syntenic homologs are added to the map. The different types of outputs are shown in the orange frames.

In order to preserve good sensitivity (*i.e*. not to lose pairs of divergent orthologs due to stringent homology criteria) and specificity (*i.e*. not to infer false homology between genes), SynChro uses two different criteria of homology to reconstruct synteny blocks between two genomes 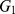 and 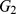. The reconstruction is achieved through three successive simple steps that are detailed in [16] and quickly recalled here (black frame in Fig. 1):

1. Identification of Reciprocal Best Hits (RBH, also called BDBH for Bi-Directional Best Hits) using Opscan (see Material and Methods). Two genes 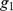 and 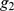, encoding two proteins 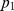 and 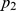 and occurring respectively in 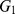 and in 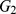, are called *RBH* if the best match of 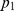 in 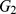 is 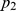 and, reciprocally, the best match of 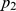 in 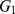 is 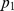. In this case, the pair of genes 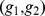, or equivalently 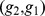, is called a RBH and 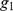 and 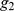 are called RBH-genes.
2. Definition of the synteny blocks. Synteny blocks are primarily defined by their anchors which correspond to series of RBH that are co-localized along chromosomes in the two compared genomes, 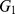 and 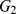. RBH are defined as anchors if they are in 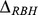 synteny. A RBH 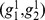 is in 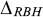 synteny with another RBH 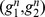 if it exists a chain of 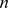 RBH 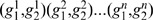, with 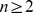, such that 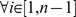 there are strictly less than 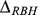 RBH-genes lying between 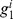 and 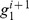 in 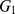 and strictly less than 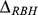 RBH-genes between 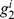 and 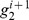 in 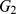. By allowing the insertion of an unlimited number of non-RBH genes, this 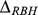 threshold allows to focus on balanced rearrangements such as inversions, translocations and chromosome fusion/fission.
3. Completion of the synteny blocks with non-RBH homologs. Two genes, 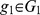 and 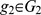, are *non-RBH homologs* (non-RBH, in short), if at least one of them does not correspond to a RBH-gene and if their amino-acid sequences share at least 30% of similarity (*i.e*. percentage of positive residues) and if the ratio between the length of the match between the two protein sequences (including internal gaps introduced by blastp) and the length of the smallest protein sequence is larger than 0.5. A pair of non-RBH 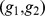 is in 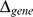 synteny with an anchor 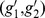, and therefore complete the corresponding synteny block, if 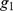 and 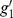 are at strictly less than 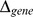 genes apart in 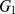, and 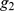 and 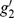 are at strictly less than 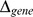 genes apart in 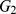. Note that in order to keep a single parameter to launch the program, called 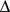, the algorithm imposes that 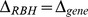 if only one value is provided by the user. Alternatively, the user can decide to provide two different values to 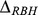 and 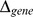. In the rest of the manuscript we will use the general 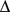 parameter to account for both 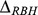 and 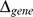.

### SynChro Input, Output and Parameter

SynChro is a set of awk and python scripts with graphical outputs supplied using gnuplot. It can be applied to two or more genomes to realize all possible pairwise comparisons.

The minimum input information that must be provided to SynChro is a list of protein-coding genes, ordered along the chromosomes (or scaffolds) and their associated amino-acid sequences. Their coordinates along chromosomes, centromere positions, and other genomic features are useful information but not compulsory for synteny block reconstruction. The indication of the coding strand is also a useful but optional information that is used to orient synteny relationships between genes in the synteny map (if they are not specified, genes are assumed to be all on the same strand). Formats of the input files are detailed in the README file (http://www.lcqb.upmc.fr/CHROnicle/SynChro.html). Allowed formats include EMBL, GenBank and Fasta files and the scripts that convert these files into the expected input format are provided within the package.

For each pairwise comparison, four different outputs are provided (see orange frames in Fig. 1):

1. a detailed synteny map allowing to visualize synteny blocks with all individual homology relationships (including their relative orientation in the two compared genomes) and the breakpoint regions including the protein-coding genes they encompass as well as other genetic features such as tRNA, pseudogene, LTR (Long Terminal Repeats), etc. This synteny map is interactive, the names of the different genetic features pop-up on the screen when the mouse points to their symbols. This map is a vectorial image, therefore it is possible to zoom in and out as necessary. This detailed synteny map represents a true improvement compared to other tools where graphical outputs are often poor, being reduced to dotplots [1], [17], [18] or chromosomal painting [19], [20].
2. text files containing homology relationships (RBH and non-RBH) and synteny blocks description
3. a chromosomal painting representation
4. a genome-wide dotplot of syntenic homologs.

Moreover, for several pairwise comparisons, SynChro provides scripts to compute, correlate and plot relevant information such as the proportion of genes/genome that is conserved in synteny, the average percentage of amino-acid similarity between orthologs, the number of synteny blocks, the average length (in nucleotides or in number of genes) of the breakpoint regions (*i.e*. regions between two contiguous synteny blocks), the average number of genes per synteny block or the proportion of consecutive synteny blocks whose homologous blocks map also on the same chromosome in the other species (see the README file for the complete list).

Another script is also provided to reconstruct families of orthologous genes (*i.e*. syntenic homologs, RBH and non-RBH, shared between multiple genomes inferred by transitivity from the pairwise relationships) containing exactly one gene per genome (all families containing duplicated genes are discarded). More formally, given a graph where vertices represent genes from multiple genomes and edges represent the RBH and the non-RBH homology relationship (deduced from all pairwise comparisons), each connected component (independent group of vertices linked together) containing one and only one gene per genome is defined as a family of orthologous genes. Families of orthologous genes could be very useful. For instance, delineating such families is of primary importance to define a set of genes that can be used in phylogenetic reconstruction.

SynChro is very easy to use as there is only one parameter to set up, the synteny block stringency 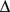. The 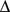 parameter is easy to learn and to master: higher values of 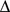 are more permissive and allow larger micro-rearrangements to be tolerated within synteny blocks while smaller values of 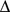 are more stringent and split synteny blocks at micro-rearrangement breakpoints. Table 1 illustrates the evolution of the number of reconstructed synteny blocks and the number of syntenic RBH involved in these blocks as a function of the 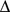 value for three comparisons: *Homo sapiens*/*Mus musculus*, *Homo sapiens*/*Taeniopygia guttata* and *Homo sapiens*/*Danio rerio*. It shows that for the two first comparisons, the number of syntenic RBH in synteny blocks do not increase drastically, confirming that the main impact of 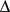 is to split, or merge synteny blocks. However, for more distantly related genomes such as in the third comparison (Human/Zebrafish), the number of syntenic RBH increases with 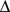, as do the number of synteny blocks, meaning that, for larger phylogenetic distances, increasing the 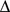 value allows, above all, to recover a larger number of synteny blocks.

**Table 1:** Evolution of the number of synteny blocks and syntenic homologs as a function of the 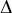 value.

	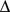	1	2	3	4	5	6	7
Human/	# synteny blocks	1 279	446	377	354	339	331	318
Mouse	# syntenic RBHs	13 786	13 995	14 031	14 035	14 045	14 047	14 054
Human/	# synteny blocks	1 217	727	654	628	604	575	555
Zebra finch	# syntenic RBHs	6 995	7 258	7 311	7 343	7 358	7 372	7 396
Human/	# synteny blocks	1 652	1 812	1 833	1861	1 868	1892	1 900
Zebrafish	# syntenic RBHs	4 206	5 157	5 542	5 791	5 970	6 152	6 317

### Benchmarking SynChro on Vertebrate Genomes

To evaluate the performance of our algorithm, we compared the synteny block reconstruction achieved by SynChro to the synteny blocks reconstructed by two other commonly used tools that also reconstruct synteny blocks from annotated genome/genes: MCScanX [14] and i-ADHoRe [15]. These tools are regularly updated since their first publication [9], [21]. The three tools were run on the same dataset composed of three pairwise comparisons of genomes corresponding to three different levels of synteny conservation: Human/Mouse (*Homo sapiens*/*Mus musculus*), Human/Zebra finch (*Homo sapiens*/*Taeniopygia guttata*) and Human/Zebrafish (*Homo sapiens*/*Danio rerio*). SynChro appears to be between 2 and 3 time faster than the two other tools to reconstruct synteny blocks between the three pairwise comparisons (SynChro takes, on a desk computer, on the order of 40 minutes to reconstruct synteny blocks between two vertebrate genomes, Table 2).

**Table 2:** Characteristics of SynChro, MCScanX and i-ADHoRe synteny blocks for three pairwise comparisons.

		SynChro	MCScanX	i-ADHoRe
Human/mouse	time (in minutes)	36 (Opscan)+9 (non-RBH+blocks)	131 (blastp)+1 (blocks)	131 (blastp) +1 (blocks)
	# blocks	339	602	497
	# syntenic homologs	25 000(14 045)	14 624(14 624)	19 349(14 205)
	% syntenic homologs	80.1	69.2	69.0
	% genome within synteny blocks	89.3	89.3	89.3
Human/Zebra finch	time	27+6	65+0	65+0
	# synteny blocks	604	552	767
	# syntenic homologs	10 833(7 358)	8 879(8 879)	10 377(9 489)
	% syntenic homologs	49.2	43.8	46.2
	% genome within synteny blocks	71.3	70.9	71.7
Human/Zebrafish	time	35+10	122+1	122+1
	# synteny blocks	1 868	627	1115
	% syntenic homologs	9 279(5 970)	3 958(3 958)	6 239(5 028)
		39.8	18.1	22.8
	% genome within synteny blocks	49.9	39.3	37.3

The execution time (in minutes) indicates the time used for homolog identification and for synteny block reconstruction (for SynChro, these two steps are not really separable because reconstruction of synteny blocks implies the identification of additional non-RBH homologs by blastp). The number of syntenic homologs represents the total number of homology relationships in the synteny blocks. The numbers between brackets indicate the number of homology relationships when only one relationship per gene per synteny block is allowed (*i.e*. removing the homology relationships corresponding to tandemly duplicated genes within a given synteny block). Note that for MCScanX these 2 values are identical because the program was run with the 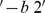 option which prevents MCScanX to detect tandemly duplicated genes within a given synteny block.

**Figure 2:**
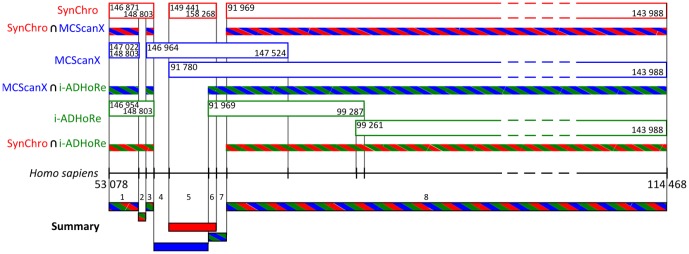
Congruence between the 3 different synteny block reconstructions. An example based on a segment of the *Homo sapiens*' X chromosome (from coordinates 53,078 to 114,468 kb) and the genome of *Mus musculus* is presented. The synteny blocks reconstructed by the three tools, SynChro, MCScanX and iADHoRe are represented by red, blue and green-framed open boxes, respectively. The two coordinates, inside each box, refer to the coordinates in the mouse genome. Synteny blocks from 2 different reconstructions are congruent when overlaping synteny blocks, along the human chromosome X, map overlapping regions in the mouse genome. These congruent synteny blocks are represented by hatched bi-colored boxes and are denoted: SynChro
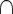
MCScanX, MCScanX
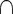
i-ADHoRe and SynChro
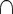
i-ADHoRe. The intersection of these three sets of synteny blocks allows to define regions (such as regions 1, 3 and 8) where the three tools are in agreements (tri-colored hatched boxes) and to deduce regions (such as the other regions) where only one or two tools detect synteny conservation (or are in agreement). The 5 lines at the bottom of the figure summarize these regions. Note that overlapping synteny blocks predicted by MCSanX or i-ADHoRe correspond to regions containing duplicated genes between the blocks. These regions do not necessarily contain many duplicated genes given that a single duplicate is sufficient to produce an overlap.

**Figure 3:**
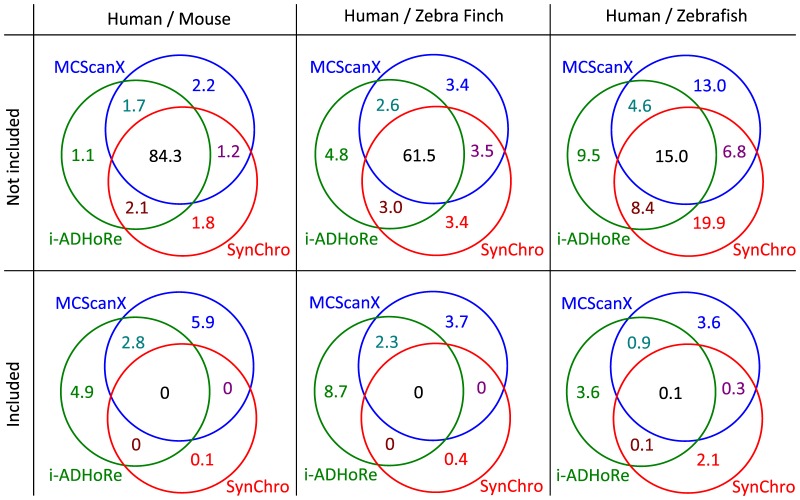
Venn diagrams showing the congruence between the three tools: SynChro, MCScanX and i-ADHoRe. The numbers indicate the percentages of the human genome found in (congruent) synteny (with the mouse, zebra finch, zebrafish genomes) by one, two or three tools. The first row, ‘Not included’, reports the proportions that are comprised within not-included synteny blocks (consecutive or partially overlapping) in the human genome, whereas the second row, ‘Included’, shows the proportions of the human genome that is recovered by included synteny blocks which mostly represent duplicated regions.

In order to quantify the level of consistency between the three tools, we compared the coordinates of the syntenty blocks detected by the different tools to quantify the proportion of the human genome that was covered by the same synteny blocks by the different tools (Fig. 2). For each pairwise comparison, this quantification was performed by scanning the human genome to identify the regions where synteny blocks from two different tools are overlapping and by checking if their homologous blocks in the other genome were also overlapping (if so, these synteny blocks are said to be congruent). Only two tools were compared at a time and then the intersection between the three two-way comparisons was realized. This analysis allowed identifying different types of regions in the human genome: regions congruently covered by the three tools, regions covered by the three tools but with some discordances (*i.e*. one or two tools would map different non-overlapping regions in the other genome), regions covered by only one tool, regions not covered by any of the three tools, etc. (in total 15 different types of regions were identified). As an example, Figure 2 shows 8 successive regions representing 6 different types. For each tool, we quantify from these regions the proportions of the human genome where synteny was supported (i) only by this tool (or also by the other tools but not consistently with the considered tool), (ii) consistently by this tool and another one and (iii) consistently by the 3 tools (see the Venn diagram, in Fig. 3). In the case of overlapping synteny blocks (as the two last blocks of MCScanX, or the two last blocks of i-ADHoRe, in Fig. 2), the region is considered to be congruent if at least one of the two overlapping synteny blocks is congruent with a synteny block detected by another tool (see the intersection SynChro
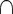
MCScanX in Fig. 2). In addition, congruence between the different tools was assessed separately for regions covered by successive or partially overlapping synteny blocks (referred as ‘Not included’ in Fig. 3) and for regions covered by synteny blocks where one block was included in a larger block (mostly representing duplicated regions and referred as ‘Included’ in Fig. 3, respectively).

From these analyses, we first estimated the proportion of the human genome that was found to be conserved in synteny by at least one of the three detection tools. This proportion cannot be directly deduced from the Venn diagram by summing up the 7 percentages because regions where two or three tools disagree (such as the regions 5 and 6 in Fig. 2) would be counted two or three times. This proportion is in fact at least equal to the highest proportion of the genome recovered by only one tool (*e.g*. at least 89.4% of the human genome was found in synteny with the mouse genome because 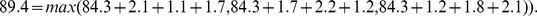 This proportion decreases with increasing phylogenetic distances between compared genomes: 89.4% between Human and Mouse, 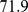
% between Human and Bird and 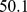
% between Human and Fish (Fig. 3, top). In the first two pairwise comparisons involving relatively close genomes (Human/Mouse and Human/Zebra finch), a large proportion of the human genome was congruently recovered by all three tools, 84.3% and 61.5%, respectively. Between 5% and 20% of the genome were recovered either by only one tool or congruently by two tools or even not congruently by two or three tools (Fig. 3). These results, and in particular the proportions specifically found by each of the three methods, show that all three tools can efficiently reconstruct synteny blocks between these genomes and that SynChro performs equally well as the two other tools. For the comparison involving more distant genomes (Human/Zebrafish), the proportions of the genome that is congruently found in synteny by the three methods is much more limited (15%). However, the proportion of the genome that was recovered by only two methods also remains limited (between 4.6 and 8.4%) which shows that the lack of congruent synteny in this comparison does not result from the inability of one tool to correctly reconstruct synteny but rather from a true loss of synteny between these genomes probably due to the accumulation of numerous chromosomal rearrangements [22]. It is interesting to note that a proportion of the human genome co-detected by SynChro and any of the two other programs (8.4 and 6.8%) is higher than the proportion co-detected by MCScanX and iADoRe (4.6%), which suggests that SynChro could be more efficient than the two other tools to detect synteny between divergent genomes (with the parameters used in this work, see Materials and Methods). The relatively high proportion of the genome only covered by SynChro synteny blocks (19.9%, Fig. 3) can be explained by the fact that 508 synteny blocks (over the 1868 identified by SynChro, Table 2) are defined by only two anchors. These small synteny blocks escape detection with MCScanX and i-ADHoRe because of the higher minimal number of anchors that is required to define a block in these programs (5 and 3, respectively). We checked whether small blocks composed of only two genes detected by SynChro corresponded mainly to false positive blocks or if they comprise true synteny information. The probability that two pairs of homologs are found by chance as direct neighbors simultaneously in two different genomes is given by the following formula 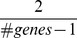. This probability equals 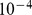 for the human genome. However, we found that 9 out of the 10 two-gene synteny blocks in the human/mouse comparison were composed of such direct neighbors (90%). For the human/bird comparison we found 26 blocks of direct neighbors out of the 42 two-gene synteny blocks (62%). For the human/fish comparison, we detected 250 blocks of direct neighbors among the 508 blocks of two genes (49%). These results clearly show that an important proportion of the small synteny blocks composed of only two genes that are detected by SynChro, do in fact comprise true synteny signal. This also suggests that the proportion of false positives in these small synteny blocks would increase for comparisons involving more remote species.

The proportion of the human genome that is comprised in included synteny blocks mainly represents the proportion of the genome that is duplicated. From the two first comparisons (Human/Mouse and Human/Zebra finch), it clearly appears that SynChro detects significantly less such regions than the two other tools (0.1 to 0.4% *vs* 3.7 to 8.7%, respectively), which was expected given that SynChro is not designed to predict duplicated regions (due to the RBH step), contrary to MCScanX and i-ADHoRe. It is noteworthy that although MCScanX and i-ADHoRe manage to detect some duplicated regions, the congruence between their predictions is rather limited (2.3 to 2.8%, Fig. 3). In addition, when a genome has undergone a recent whole genome duplication event, as it is the case for the zebrafish genome, SynChro manage to identify a non-negligible fraction of the duplicated regions (2.1% compared to 3.6% for the two other tools).

It is also interesting to note that SynChro detects more syntenic homologs than MCScanX or iADHoRe. For instance, the three tools detect an identical proportion of the genome that is conserved in synteny between Human and Mouse (89.3%, Table 2). However, both the number and the percentage of syntenic homologs in the synteny blocks are much higher for Synchro than for the two other tools (Table 2). Finally, we made the intriguing observation that the number of synteny blocks detected by MCScanX does not increase with increasing phylogenetic distances as it is expected from an increasing number of chromosomal rearrangements and as it is found to be the case with both SynChro and i-ADHoRe (Table 2).

## Materials and Methods

For each tool, the same parameters were used for the three comparisons: *Homo sapiens*/*Mus musculus*, *Homo sapiens*/*Taeniopygia guttata* and *Homo sapiens*/*Danio rerio*. The four genomes were downloaded from the *Ensemble* website (http://www.ensembl.org/info/data/ftp/index.html).

### SynChro

The RBH identification is achieved with OPSCAN (http://wwwabi.snv.jussieu.fr/public/opscan/), which is based on the FASTA algorithm [12]:

1. For each protein sequence from the query genome, OPSCAN scan the database with a simple version of the fastp algorithm where no gap is allowed and where the alignment is achieved through shifting sequences to maximize the number of matches between the two compared sequences. This step leads to the identification of a set of K most similar genes with K = 6 (default value). Other parameters are used with their default values (kuple: 2, fastp diag integ: 0 and fastp lower threshold: 5).
2. For each query gene OPSCAN refines the alignment with its K most similar target genes by performing a dynamic programming alignment (with zero cost end gaps). The parameters used are BestFit (local) and BLOSUM60 scoring matrix.
3. RBH are defined from these refined alignments when the most similar gene to the query gene Gi amongst its K most similar database genes is Gj, and the most similar gene to the database gene Gj is the query gene Gi. The parameters used are Bestfit score threshold for “homologs”: 40 (in the 0–100 range) and length ratio threshold (longest sequence divided by the shortest): 1.3.

The reason why we chose to use OPSCAN rather than blast is because this algorithm was optimized for RBH identification. The fastp part permits OPSCAN to quickly scan the database genome (by simply shifting the compared sequences), as a pre- filter for possible RBH, and then, the BestFit algorithm is run only between query genes and their K = 6 most similar homologs. OPSCAN takes only 36 min (on a desk computer) to identify RBH between the human and the mouse genome. By comparison, a single pass of blastp using the human genome as query against the mouse genome takes 131 min and it would be necessary to run blast in the other direction using the mouse genome (or a subset of it) as query to identify RBH.

There is only one parameter to set up in SynChro, the synteny block stringency 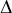. This parameter concomitantly sets both the 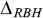 and the 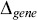 parameters although each value can also be set separately (see the description of the SynChro algorithm above). The same value 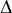 = 5 was used for the three pairwise comparisons of genomes described in this work. This 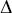 value allows a maximum of 4 intervening RBH within a synteny block which is well-suited to identifying synteny blocks between human and zebrafish (even if Table 1 shows that other values would have been fine too: there are no major differences between 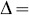
2, 3, 4, 5, 6 or 7).

SynChro uses several other parameters (% of similarity between homologs, length of the alignments, minimal number of anchors per block (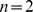, this value has no relationship whatsoever with the 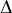 value) that have fixed values. These values were shown to be well suited to perform efficient synteny block reconstruction between a large range of organisms sharing various phylogenetic relationships (successfully applied to 18 yeast and 13 vertebrate genomes [21]). Nevertheless, the user can easily change the values of these parameters in the source code (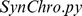) where they are clearly commented at the top of the file.

### MCScanX

MCScanX uses as input a file containing pairwise homologous relationships (typically an all-against-all BLAST search). The blastp minimal expectation value (E) was set to 1e−10 (as suggested in the manual). This value impacts the number of reconstructed synteny blocks. In addition, at least 6 parameters need to be set (even if, many of them can be used with their default value):

1. MATCH_SCORE, a final score used to validate a synteny block: we used the default value (50)
2. GAP_PENALTY, we used the default value (−1)
3. MATCH_SIZE, a number of genes required to call a collinear block: as SynChro performs synteny block reconstruction from 2 anchors, we set this parameter to the minimum (*i.e*. 5, the default value)
4. E_VALUE, the synteny block alignment significance: we used the default value (1e−10)
5. MAX_GAPS, the maximum of gaps allowed: default value is 25, which is too much permissive (each regions map tens of regions in the other genome), we used a value of 10 instead.
6. OVERLAP_WINDOW, the maximum distance (in number of genes) to collapse BLAST matches: we used the default value (5).

### i-ADHoRe

i-ADHoRe takes, as input a file containing pairwise homologous relationships (typically an all-against-all BLAST search), so we use the same e-value of 1e−10 that for MCScanX (that is why the execution time, in Table 2, corresponding to the execution of blastp, is the same for MCScanX and i-ADHoRe). To run i-ADHoRe, at least 5 additional parameters need to be set:

1. prob_cutoff, indicating the maximum probability for a cluster to be generated by chance: we use the suggested value (0,001)
2. gap_size, indicating the maximum (pseudo-)distance that should exist between points in a cluster: we use the value given as an example (15)
3. cluster_gap, indicating the maximum (pseudo-)distance that should exist between individual base clusters in a cluster: we use the value given as an example (20)
4. q_value, indicating the minimum 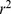-value (a measure for the linearity of a series of points) a cluster should have: we use the value given as an example (0.9)
5. anchor_points, the minimum number of anchor points: as SynChro reconstruct synteny blocks from 2 anchors, we set this parameter to the minimum, meaning 3 (the suggested values was comprised between 3 and 6)

## Conclusion

We showed in this work that SynChro is a fast, efficient and user-friendly tool to reconstruct synteny blocks between (complex) genomes harboring different levels of synteny conservation. Despite a very simple algorithm, the reconstruction is highly congruent with reconstructions obtained with more sophisticated tools. The main advantages of SynChro are the following: (i) it is fast (it takes, on a desk computer, on the order of 40 minutes to compare two vertebrate genomes); (ii) it is easy to use (a unique parameter 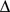, which is really simple to handle, needs to be set) and (iii) it provides a rich set of graphic outputs (notably an interactive synteny map that allows zooming in breakpoint regions).
